# Some Conditions of Artificial Stumps

**Published:** 1918-08

**Authors:** 


### ORTHOPEDICS

***Some Conditions of Artificial Stumps.*** By E. Muirhead Little. *American Journal of Orthopedic Surgery,* April, 1918.

It is suggested that those surgeons who see the end results of amputations — six months or more after operation at front hospitals, are doing a service to their colleagues by criticizing their results. Little sees many end results at Rochampton, England, to which patients are referred only after it has been certified that stumps are ready for the fitting of an artificial limb.

In a memorandum on amputations and amputation stumps, issued by the War Office in March 1916, the following are given as the requirements of a good stump:

1. A good covering for the bone;
2. Sound healing;
3. Painlessness;
4. Freedom of movement.

The following conditions were said to prevent or delay the fitting of an artificial limb.

1. Sinuses;
2. Painful nerves or tenderness due to inflammation of the bone;
3. Unsound scars;
4. Contracture in the neighborhood of the joint immediately above the amputation.

Little considers the above points seriatim. Most of them require little elaboration or comment. Painful nerves may usually be avoided by carefully searching for all nerve ends at the time of amputation, pulling them down, and cutting them as short as possible. The tenderness of a bulbous nerve is probably due to a septic neuritis, and a removal of the bulb followed by aseptic healing usually does away with the painful symptoms. Occasionally, however, the newly formed bulb becomes hypersensitive. Prolonged physiological rest often helps these cases. In a few cases the trouble is of central origin.

Not only main trunks but also the branches of cutaneous nerves may be involved. Ulcerating or badly nourished scars can be treated only by the removal of the scar and, if necessary, of enough bone to bring the skin edges together easily. Any considerable contracture is a marked drawback. It is especially objectionable and common in the hips, and its occurrence is fostered by the habit of supporting the thigh stump on a pillow for the patient's comfort. This is often unavoidable, but, if care be taken to use passive extension at the hip daily as soon as wound conditions permit, much trouble may be prevented.

As to the best forms of stumps : the functions of a limb are the influencing factors in determining the kind of stump sought. In the lower extremity the problem is simpler than in the upper, as the main function here is to support the weight of the body at rest and in motion, and to act as the propelling force in the process of locomotion. The strains to which the stump is exposed are the pressure on the end from the weight of the body, and the leverage in moving the artificial limb in locomotion. The upper extremity on the other hand has to perform highly coordinated and complicated motions. These general considerations must always be kept in view.

Generally speaking, the longer a stump the better for the prothesis; the covering should be soft and movable, and the scar should not be adherent to the bone, nor exposed to pressure. The bone should not inflict injury to soft parts when they are pressed against it. Theoretically exarticulation would appear best, but except in the cases of the hip and the shoulder, where there is no choice, exarticulation stumps are not satisfactory : 1. because the articular end of the bone is too bulky; 2. the joint of the artificial limb, unless it is placed on the sides, makes the joint much larger than the normal one and therefore very unsightly; 3. large flaps are needed, and such are often difficult to obtain and prone to slough. In war work osteoplastic resections are generally out of the question because of sepsis. Even without their aid one ought to be able to obtain 50 to 60 o/o of good end bearing stumps.

The position of the scar is very important; and a posterior scar is preferred in leg and thigh amputations. In the upper extremity non-adherent end-scars are preferable.

The guillotine amputation was originally intended as a temporary measure, reamputation being expected later on. However, healing sometimes occurs without reoperation. It is important to remember that unless the end of the bone is covered by normal healthy skin, and not by scar tissue, reamputation is advisable, and this should not be done prematurely, but only when the parts are in condition to obtain primary union.

As to formation of flaps and position of scars in the various amputations, an abstract of Little's article is impossible. Some important facts to remember are :

1. That in humerus amputations every inch of length gained is an advantage, even if the end of the bone is uncovered, as the artificial limb does not exert end pressure as in the lower extremity.

2. If one cannot get a satisfactory stump below the elbow, it is advisable to amputate just above the condyles. Short forearm stumps are very poor. The best forearm stump is one following amputation at the junction of the middle and lower thirds.

3. As a result of recent improvements, a man is better off with an exarticulation at hip (or amputation through neck of femur) than he is with a stump of six inches of femur.

4. The best thigh amputation is through the lower third: if possible a long hooded flap should be made, giving a posterior scar.

5. For the leg, Little recommends amputation below the middle, though the consulting surgeons at Rochampton recommended a site four inches below the knee joint.

6. When necessary to go below the junction of middle and lower thirds of leg, a Symes amputation gives the best stump. If the metatarsus cannot be saved, it is not worth while to preserve the tarsus. The writer prefers an atypical Symes, dividing the bones well above the malleoli instead of at the usual level, thus avoiding a bulky stump. He also advises making the section at right angles to the axis of the whole leg instead of the lower fourth avoiding a troublesome varus. Care should be taken to remove the plantar nerves from the flap or to resect the posterior tibial nerve. One should never fail in any leg amputation to section the fibula at least one half inch higher than the tibia.

